# Elevated 12-Month and Lifetime Prevalence and Comorbidity Rates of Mood, Anxiety, and Alcohol Use Disorders in Chinese Men Who Have Sex with Men

**DOI:** 10.1371/journal.pone.0050762

**Published:** 2013-04-18

**Authors:** Lianzheng Yu, Chao Jiang, Jun Na, Ning Li, Wenli Diao, Yuan Gu, Li Zhao, Yan Zou, Ying Chen, Li Liu, Huijuan Mu, Yunyong Liu, Liya Yu, Xiaoli Yang, Guowei Pan

**Affiliations:** 1 Department of Chronic Diseases, Liaoning Provincial Center for Disease Control and Prevention, Shenyang, China; 2 Department of Epidemiology, Dalian Medical University, Dalian, China; 3 Department of AIDS, Shenyang Municipal Center for Disease Control and Prevention, Shenyang, China; 4 Department of AIDS, Anshan Municipal Center for Disease Control and Prevention, Anshan, China; 5 Department of AIDS, Dandong Municipal Center for Disease Control and Prevention, Dandong, China; 6 Department of AIDS, Benxi Municipal Center for Disease Control and Prevention, Benxi, China; 7 Department of Mental Health, Shenyang Medical College, Shenyang, China; University of Iowa Hospitals & Clinics, United States of America

## Abstract

**Background:**

This study aimed to assess whether Chinese men who have sex with men (MSM) had a significantly elevated prevalence of psychiatric disorders compared to urban males in China.

**Methods:**

807 MSM were recruited using a respondent-driven sampling (RDS) method in urban area of northeast China. Psychiatric disorders were assessed employing the Composite International Diagnostic Interview (CIDI. Version 1.0) according to the criteria of the DSM-III-R.

**Results:**

Chinese MSM had a significantly elevated standardized prevalence ratios (SPR) for lifetime prevalence of any disorder (SPR = 2.8; 95%CI: 2.5–3.2), mood disorder (SPR = 3.0; 95%CI: 2.3–3.7), anxiety disorder (SPR = 5.5; 95% CI: 4.6–6.5), alcohol use disorder (SPR = 2.4, 95%CI: 2.0–2.8), and combination of disorders (SPR = 4.2; 95%CI: 3.4–5.1).

**Conclusions:**

Chinese MSM had significantly elevated prevalence and comorbidity of psychiatric disorders. RDS is a suitable sampling method for psychiatric epidemiological survey in MSM population.

## Introduction

Men who have sex with men (MSM) have long been known to exist in China, but were rarely recognized or accepted by the general population [1]. However, this hidden population has received increasing attention in recent years because of a high HIV infection rate [2]. About 11.0% of 700 000 Chinese HIV/AIDS patients are MSM, and acquired the disease by homosexual transmission [3]. The total population of Chinese MSM has been estimated at 17.82 (10.18–25.45) million [4]. With the increasing recognition of sexual diversity and the rise in personal freedom over the past three decades, Chinese MSM have emerged as a less invisible group in society, and their existence has become gradually recognized. However, tolerance of homosexuality by non-homosexuals has remained low. Several studies showed that Chinese MSM encountered discrimination and victimization, and experienced confusion, a need to hide their behavior, and a sense of shame [5], [6]. A national survey showed that 34.5% and 10.6% of Chinese gays or bisexuals had attempted or committed suicide, respectively [7]; several major studies out of China have shown that MSM are particularly vulnerable to psychiatric disorders, compared with heterosexual males [8], . And a recent systematic review showed that gay and bisexual males experienced at least a 1.5-fold excess of depression, anxiety, and alcohol use disorders, compared to their non-gay counterparts [10]. However, no study to date has used an internationally standardized instrument to evaluate the prevalence of psychiatric disorders among the Chinese MSM population. As MSM constitute a hard-to-reach or hidden population, it can be difficult to survey MSM using standard sampling methods because the requisite sampling frames do not exist [11]. A population-based survey that includes questions on the gender of sexual partners, such as the National Comorbidity Survey (NCS) employed in the United States [9], can compare mental health status between individuals of different sexual orientations without encountering the usual problems of sampling bias or the absence of heterosexual reference groups. However, the extremely large sample size required makes such surveys difficult to conduct because of the considerable financial and personnel resources required [12]. Institutional and time-location sampling methods have been employed to reach such hidden populations, but difficulties in the control of bias may cause the results to be unrepresentative [13]. Respondent-driven sampling (RDS), a network-based technique used to estimate traits in hard-to-reach populations, is now widely employed in the public health sector and has been used in more than 120 studies conducted in more than 20 countries [14]. However, very few reports on the application of this survey modality in the field of mental health have appeared. To assess whether Chinese MSM had a significantly elevated level of psychiatric disorders, as reported in MSM of other countries, we surveyed 807 MSM using a respondent-driven sampling (RDS) method in four cities of Liaoning province, northeast China.

## Methods

### Methods for recruitment

The target population was MSM residing, working, or living in four cities of Liaoning province (Anshan, Benxi, Dandong, and Shenyang). To be eligible for inclusion in the study, each respondent had to report oral or anal sexual intercourse with another man in the past 12 months; to be aged between 18 and 65 years; to agree to complete a questionnaire and to give blood for testing; to have a recruitment card; and not to be a repeated participant (controlled by daily updating of a fingerprint recognition system). At least 200 MSM were required to be recruited in each city. The study protocol was reviewed and approved by the Institutional Review Board of the Liaoning Provincial Center for Disease Control and Prevention (CDCP).

We used RDS to recruit study participants [15]. Briefly, we selected 5–10 “seeds”, heterogeneous by age and the sites at which they found sexual partners (bars, bathhouses, gardens, via the internet, and public toilets) in each city, and gave each seed three coded coupons to pass on to his sexual partners. MSM recruited by seeds were asked to recruit the next wave of respondents, and the process continued until the target sample size was achieved. Participants in each city were told in advance that recruitment would stop when the desired sample size was attained. The survey was conducted in the municipal CDCPs of the four cities. All survey sites were open on weekends and/or during evening hours to accommodate those who were unable to participate during weekdays. All participants, regardless of whether or not they agreed to serve as recruiters, were paid 80 *yuan* for participation in the study. Recruiters received an additional 10 *yuan* for each eligible person (up to three in number) whom they recruited. After complete description of the study to each respondent, written informed consent was obtained. Most interviews were digitally recorded to control the quality of investigation. If respondents refused to be recorded during interview, we recorded their refusals. Age, socio-demographic characteristics, sexual orientation, and education level, were noted, using a structured questionnaire. Data on personal network size, and the nature of the relationship with the recruiters (information specifically required for application of RDS methodology), were also collected. No name or other personal information, such as an identification number, was recorded.

### Quality control

The interviews were conducted by 20 public health doctors from Liaoning provincial CDCPs and 4 from municipal CDCPs. The doctors had an average of 7 years of prior interviewing experience in various investigations; 8 had used the CIDI. Version 1.0 questionnaire during the mental health survey conducted in Liaoning province in 2004 [16]. In addition, all interviewers received 7-day study-specific training in the use of CIDI1.0, conducted by psychiatrists from China Medical University and Dalian Psychiatric Disorder Hospital, who had been trained by a WHO authorized trainer in World Mental Health survey methodology, in Beijing [17]. Quality control protocols were standardized across cities to check on the accuracy of interviewers. Four regional supervisors from Liaoning CDCP, working in the field, checked each completed interview to detect missing data and unclear responses; incomplete questionnaires were returned to the interviewer, who contacted respondents to obtain the missing information. At least 10% of digital voice recordings were randomly checked by each field supervisor to verify the acceptability of interviewer performance. If any problem was found, the respondent was re-contacted by the field supervisor.

### Measurement and evaluation techniques

We used a Chinese version of CIDI1.0 to conduct face-to-face interviews [18]; this instrument has shown good reliability and validity [19], and has been employed in China in several epidemiological surveys of psychiatric disorders conducted during the past decade [16], [17]. The instrument is a fully structured diagnostic modality designed to be used by trained interviewers who are not clinicians. CIDI1.0 generates diagnoses according to both the ICD –10 and DSM-III-R diagnostic criteria; DSM-III-R diagnoses were used in the current report. Core conditions included mood disorders (major depression, dysthymia, and bipolar disorder); anxiety disorders (panic disorder, generalized anxiety disorder, simple phobia, social phobia, agoraphobia, and obsessive-compulsive disorder); and substance use disorders (alcohol and drug abuse and dependence). Diagnoses were made without the use of diagnostic hierarchy rules [20], meaning that an individual could meet the criteria for any disorder regardless of the presence of other disorders.

### Statistical Analysis

We used the Respondent-Driven Sampling Analysis Tool (RDSAT, free available at http://www.respondentdrivensampling.org/) to analyze data; the program adjusts for use of the long-chain referral recruitment design to produce population estimates. Crude sample estimates (*i.e.*, the proportions of key variables in the sample per se) were adjusted to reflect the makeup of the target MSM population, based on tracking of who recruited whom and the relative sizes of the networks of the various participants. RDSAT allows use of data to create individualized weights based on the characteristics of those who referred others to the study. When we imported the vector of individualized weights into a SAS program, we could reproduce the population estimates yielded by RDSAT [21]. To eliminate outliers of network size, we set the RDSAT size for pull-in outliers to 1% when calculating individual weights.

We calculated “tolerance”, defined by Heckathorn [15] as the mean absolute discrepancy between an actual final sample composition and the RDS-estimated sample equilibrium composition, to assess any bias that was possibly introduced by the non-random seed selection method. Age group, marital status, income level, self-reported level of sexualorientation, and years of education were selected as trait features for composition convergence analyses using RDSAT; a tolerance of 0.02 or less indicated that actual sample compositions had converged to reach equilibrium, and population proportions were thus unbiased by non-random seed selection.

Because almost the same numbers of MSM were sampled in each of the four cities which, however, differ in population size, there was a tendency toward oversampling in smaller cities and undersampling in the larger. Therefore, the distribution of age-group of 2008 of Liaoning province was used to calculate a city/age-group-specific population weight for each subject, which was the inverse of the selection probability of each age group assigned to a sampled city, defined as:


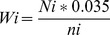
 Where Wi was the weight of the i^th^ age group, Ni was the number of males in the general population of the i^th^ age group of 2008 in each city, and ni was the number of MSM sampled in the i^th^ age group. We do not know the exact numbers of MSM in the sampled cities. This was estimated by multiplying Ni by 3.5%, the approximate mean MSM proportion (3–4%) among males in China [4], under the assumption that the proportions of MSM aged between 18 and 65 years would be consistent across each age group. Next, we calculated overall weights by multiplying individualized weights generated by RDSAT by the city/age-group-specific weight for each subject, and used these values to estimate weighted 12-month and lifetime prevalence values of DSM-III-R disorders in the general target population (*i.e.*, urban MSM in Liaoning province). We found no significant difference between prevalence when seeds were included or excluded, and thus present the former dataset in this report.

We used the weighted prevalence of corresponding psychiatric disorders in regional urban males as reference values [16]; these were obtained by the present authors, using the same CIDI1.0, in 2004. There were 2587 respondents, male and between 18–65 years-old, in the reference group. The sample weights adopted for reference groups were defined as: Wi_re_ = Ni_re_/ni_re_, where Wi_re_, Ni_re_, and ni_re_ were defined in a manner similar to Wi, Ni, and ni of the MSM group.

To determine if MSM were at an elevated risk of mood, anxiety, or alcohol disorder, or a combination thereof, we used standardized prevalence ratios [22] and 95% confidence intervals (95% CIs). Among MSM, the expected number of psychiatric disorders in each age-specific group was calculated by using the prevalence of each age-specific group from the population-based controls [16]. The total number of expected psychiatric disorders among MSM was calculated by summing the numbers of psychiatric disorders expected in all age-specific groups. The ratio of the observed number of MSM with psychiatric disorders to the expected number was defined as the standardized prevalence ratio (SPR). We assumed that the data would have a Poisson distribution; therefore, the confidence intervals for the SPRs were estimated using standard errors. Comorbidity was identified when a subject experienced more than one of mood, anxiety, and alcohol use disorders.

## Results

A total of 807 MSM, including 31 seeds, were recruited; 206, 201, 200, and 200 in Anshan, Benxi, Dandong, and Shenyang, respectively. Among the 31 seeds, 22 (71.0%) fulfilled their quotas (*i.e.*, provided three recruits), and 6 (19.4%) and 23 (74.2%) of the referral chains commenced with two or three “branches”. Twenty-three (74.2%) seeds were the originators of four or more recruitment waves, which produced 712 recruits, accounting for 91.8% of all those interviewed. The sample composition within each wave gradually changed, and became stabilized at about 4–5 waves.

The average age of the 807 MSM was 27.2±0.3 years; 68.2% were under 30 years of age, 15.2% were married, 12.3% were cohabited with men, 46.4% were bisexual, 38.8% were gay, and 12.8% were of unknown sexual orientation(Table 1). All tolerance values were lower than the cutoff value (0.02).

**Table 1:** Demographic characteristics and an examination of equilibrium.

Characteristic	Unweighted		Weighted a		Tolerance b
	N	%	N	%	
Age, years					
188–29	575	71.3	550	68.2	0.003
308–39	133	16.5	132	16.3	
408–64	99	17.3	125	15.5	
Marital status					
Single	571	70.8	547	67.8	0.002
Married	108	13.4	123	15.2	
Cohabiting	92	11.4	99	12.3	
Divorced	36	4.5	38	4.7	
Income level					
Low	305	37.8	318	39.4	0.002
Middle	280	34.7	291	36.1	
High	222	27.5	197	24.5	
Sexual orientation					
Gay	381	47.2	313	38.8	0.001
Heterosexual	13	1.6	17	2.1	
Bisexual	325	40.3	374	46.4	
Unknown	88	10.9	103	12.8	
Education, years					
<10	255	31.6	287	35.6	0.006
108–12	322	39.9	300	37.2	
≥13	230	28.5	220	27.2	

a Weighted by individual weights.

b Tolerance: Weighted mean absolute discrepancy between actual and equilibrium sample composition.

The adjusted 12-month and lifetime prevalence rates of any psychiatric disorder were 27.5% and 32.3% for MSM, 3.0 and 2.8 times the rates of the general population of urban males, respectively (Table 2). MSM were more likely to experience a combination of two or more disorders in the past year or during their lifetime. Alcohol use disorder showed the highest 12-month and lifetime prevalence rates (17.4% and 18.9%, respectively), followed by anxiety (13.7% and 18.6%) and mood disorders (5.1% and 11.7%). However, anxiety disorder showed the greatest 12-month and lifetime SPR values (6.6 and 5.5, respectively), followed by those for mood and alcohol disorders. Alcohol dependence, agoraphobia, simple phobia, and alcohol abuse were the top four individual disorders showing the highest 12-month and lifetime prevalence values. Panic disorder, simple phobia, bipolar disorder, and agoraphobia were the top four individual disorders yielding the highest SPRs for 12-month prevalence; whereas panic disorder, social phobia, bipolar disorder, and agoraphobia were the top four individual disorders showing the highest SPRs in terms of lifetime prevalence.

**Table 2:** Twelve-month and lifetime adjusted prevalence and standardized prevalence ratios (SPRs) of DSM-III-R disorders.

Disorder	Twelve-month prevalence							Lifetime prevalence						
	MSM		Urban males		SPR	95% CI		MSM		Urban males		SPR	95% CI	
	N	%	N	%				N	%	N	%			
Mood disorder	53	5.1	62	2.2	2.5	1.8	3.5	92	11.7	96	3.5	3.0	2.3	3.7
Major depression	29	2.4	48	1.6	2.1	1.3	3.2	55	6.5	75	2.8	2.2	1.6	2.9
Dysthymia	13	0.7	30	1.1	1.7	0.9	3.0	28	3.7	54	2.2	2.4	1.6	3.4
Bipolar disorder	15	2.3	5	0.2	5.2	2.2	10.3	19	2.6	7	0.2	6.1	3.1	10.9
Anxiety disorder	122	13.7	70	1.9	6.6	5.3	8.0	169	18.6	123	3.7	5.5	4.6	6.5
Panic disorder	12	1.7	5	0.1	16.1	6.4	33.4	14	2.0	6	0.1	12.1	4.9	24.5
GAD	5	0.3	10	0.2	2.6	0.8	6.2	6	0.8	16	0.4	1.2	0.5	2.6
Simple phobia	49	6.7	33	0.7	4.1	2.7	5.9	69	8.5	66	1.8	6.0	4.6	7.6
Social phobia	30	3.4	21	0.5	4.5	2.9	6.7	41	5.2	23	0.6	5.7	3.9	8.0
Agoraphobia	62	6.8	34	1.2	7.1	5.4	9.0	93	9.1	54	1.9	6.1	4.9	7.5
Alcohol disorder	157	17.4	191	7.5	2.6	2.2	3.2	167	18.9	238	9.2	2.4	2.0	2.8
Alcohol abuse	34	5.5	73	2.7	1.7	1.1	2.5	43	7.1	102	3.7	1.6	1.1	2.2
Alcohol dependence	123	12.1	118	4.9	3.2	2.6	3.9	124	12.2	136	5.5	2.9	2.4	3.6
Only one disorder	161	17.8	224	9.0	2.7	2.3	3.3	166	16.9	287	10.9	2.3	1.9	2.7
Two or more disorders	78	9.1	56	1.6	4.0	3.0	5.1	118	15.4	93	3.2	4.2	3.4	5.1
Any disorder	239	27.5	280	10.6	3.0	2.6	3.5	284	32.3	380	14.1	2.8	2.5	3.2

GAD: Generalized anxiety disorder.

Table 3 compares comorbidity status in terms of the three categories of psychiatric disorder evaluated, between 239 MSM and 284 regional urban males diagnosed with DSM-III-R conditions. MSM were about 2-fold more likely to experience two or more disorders in the past 12 months than were urban males (29.2%; 14.2%). This was also true of the lifetime prevalence of disorders (37.6%; 17.9%), especially when the high-level co-occurrence of three disorders in the past 12 months (9.6%; 1.1%) and over the lifetime (13.0%; 2.4%) were considered.

**Table 3:** Comorbidity of Psychiatric disorders among MSM and urban males during the past 12 months/lifetime.

Disorder	Twelve-month				Lifetime			
	MSM		Urban males		MSM		Urban males	
	N	%	N	%	N	%	N	%
Single disorder	169	70.7	171	85.8	81	62.3	312	82.1
Mood	11	4.6	33	11.8	18	6.3	43	11.3
Anxiety	60	25.1	36	12.9	78	27.5	69	18.2
Alcohol use	98	41.0	171	61.1	81	28.5	200	52.6
Comorbid disorders	239	29.2	40	14.2	207	37.6	68	17.9
Mood & anxiety	11	4.6	20	7.1	21	7.4	30	7.9
Mood & alcohol use	8	3.3	6	2.1	16	5.6	14	3.7
Anxiety & alcohol use	28	11.7	11	3.9	33	11.6	15	3.9
Mood & anxiety & alcohol use	23	9.6	3	1.1	37	13.0	9	2.4
Total	239	100.0	280	100.0	284	100.0	380	100.0

## Discussion

We employed a standardized RDS sampling procedure to examine the prevalence rates of psychiatric disorders, and combinations thereof, in Chinese MSM. Sample composition stabilized after about 4–5 waves, in line with the hypothesis that the recruitment system followed a first-order Markov process [15]. All tolerances for trait variables were less than 0.02, indicating that the sampled MSM produced unbiased estimates for the target population, (*i.e.*, the urban MSM of Liaoning province). Several recent studies have reported that challenges were encountered when RDS was used. The problems included high variance, narrow confidence intervals for the estimates made, and over-or under-representation of some variables [238–25]. A latest study shows that RDS may yield estimates that are either modestly better than, or in fact potentially superior to, those afforded by convenience sampling [25]. Consistent with results obtained when RDS was applied in other studies on MSM of China [2], [5], we found that RDS was reliable when used to survey the prevalence of psychiatric disorders in Chinese MSM. Few sampling methods were suited for a hidden population, such as MSM. RDS used peers or respondents to find out this population. It meets the first need of sampling a hidden population. Institutional and time-location sampling (TLS) method was another available sampling measure for MSM. But difficulties in the control of bias may cause the results to be unrepresentative [13]. And compared with TLS, using RDS, survey staff typically works from fixed sites where their physical security can be guarded. And the fixed sites facilitate conducting biological testing and relevant counseling and provision of other prevention, care and treatment services at the time of the survey.

Several studies have confirmed the validity and reliability of the Chinese versions of CIDI1.0 [19] and 3.0 [26], which have been used in several epidemiological surveys of psychiatric disorders in China over the past decade [16], [17], [27]. Most interviewers who participated in the present work had experience in the use of CIDI1.0 during a previous mental health survey conducted in Liaoning province [16], and all received a 7-day CIDI1.0 study-specific training program. A standardized quality-control procedure was in place across the four cities. These features caused the results of our investigation to be reliable and comparable among cities.

The World Mental Health Survey (WMH) showed that the lifetime prevalence of any WMH-CIDI/DSM-IV disorder in Chinese adults in Beijing and Shanghai was 13.2%, only 30–50% of the levels seen in the United States and other Western countries, China is ranked as the country with the lowest prevalence of psychiatric disorders [17]. A recent study conducted in four provinces of China found that the 1-month and lifetime prevalence of psychiatric disorders was 13.3% and 15.8% (excluding the disorders of not otherwise specified, NOS), respectively, confirming the relatively low level of psychiatric conditions in Chinese adults [28]. The 12-month and lifetime prevalence of any DSM-III-R disorder in urban males of Liaoning province was 8.9% and 12.3% [16], respectively, consistent with the above findings. However, the large background difference in the prevalence of psychiatric disorders between Chinese and other populations must be borne in mind when the prevalence and severity of such conditions are compared between MSM in China and other countries.

The prevalence of any psychiatric disorder was substantially different when homosexual and heterosexual men were compared [10]. The 12-month and lifetime prevalence rates of any DSM-III-R disorder in Chinese MSM (27.5% and 32.3%, respectively) were lower than the values found in most population-based studies among homosexual men in Western countries [8, 9, 298–31], being higher only than those of Latino and Asian-American gay or bisexual subjects (15.6% and 25.4%, respectively) [32], and close to the 12-month prevalence (28.8%) noted in the 1996 National Household Survey of Drug Abuse in the United States [33], in which the prevalence rates of only six psychiatric disorders were assessed. The relatively low incidence of psychiatric disorders in homosexual men in both China and Asia/America is in line with the low prevalence of such conditions in the general population of China [16], [17], [28].

A few studies in the United States [298–31] found that MSM had at least a 2-fold elevated risk of any psychiatric disorder, compared to their heterosexual counterparts, and a recent meta-analysis of studies on lesbians, gays, and bisexuals (LGB) found a 2-fold elevated risk of experience of a 1-year (OR  = 2.03, 95% CI  = 1.68–2.46) or lifetime (OR  = 2.41, 95% CI  = 1.91–3.02) disorder [34]. However, some reports from the United States [9], [33] and the Netherlands [8] did not find a significantly elevated prevalence of such disorders in these groups. We found a 3-f old elevated 12-month (SPR  = 3.0, 95% CI  = 2.6–3.5) and lifetime (SPR  = 2.8, 95% CI  = 2.5–3.2) prevalence of any disorder, in the present study. In contrast to what was noted in Asian-American gays and bisexuals, our results confirm the hypothesis that Chinese MSM are at significantly elevated risk of psychiatric disorders, as are MSM of Western countries, although the absolute disorder prevalence rates were relatively low in the present study.

As with any disorder studied, the prevalence of anxiety in both Chinese MSM and the general population was significantly lower than that of their counterparts in the Netherlands [8] and the United States [9], [31], especially in terms of lifetime prevalence. However, the elevated risks of 12-month (SPR  = 6.6, 95% CI  = 5.3–8.0) and lifetime (SPR  = 5.5, 95% CI  = 4.6–6.5) prevalence rates for anxiety were 2-to 3-fold those of MSM in the Netherlands [8] and the United States [32], and two meta-analyses found that the frequency of any anxiety disorder was elevated by less than 2-fold in MSM [10] and LGB [34]. The 12-to 6-fold elevation in the prevalence of panic disorder and agoraphobia noted in the present work is in line with the 6-to 4-fold elevation in the risks of panic disorders and agoraphobia in the United States [10], 34 and the Netherlands [8]. The 5-to 6-fold excess in the prevalence of social phobia is about 3 times that of MSM in the Netherlands [8]. The significantly elevated prevalence of some anxiety disorders may indicate that Chinese MSM experience more fear than distress; a genetic or constitutional factor may relieve environmental stressors. Alternatively, the coping skills needed to deal with stress may be less developed in Chinese men [35]. A recent study in Shanghai city showed that most Chinese MSM kept their homosexuality secret, to avoid becoming socially ostracized, with loss of support and respect [6]. Only 15.4% of the MSM of the present study had revealed their sexual orientation to friends/family members, suggesting a low tolerance for homosexuality and/or some specific risk factors for gays in Chinese society. A homosexual lifestyle without marriage, and being childless, are considered shameful, selfish, and unacceptable to the family and society in traditional Chinese culture. Moreover, familial pressure has become even greater upon introduction of the one-child-one-couple policy in China [6]. Further study of this aspect of Chinese attitudes is warranted.

As with anxiety disorders, the 12-month and lifetime prevalence of mood disorders in Chinese MSM and urban males were only about 25–30% those of their counterparts in the Netherlands [8] and the United States [17], [29], but the elevation in risk was similar, being 2-to 3-fold. This is close to the 2-fold elevation seen in LGB, as reported in a Meta-analysis [34], but higher than that noted in Latino and Asian-American homosexual men [32]. The significant elevation in mood disorders apparent in the present work may be associated with the low tolerance toward homosexuality shown by Chinese society, and the stigma, discrimination, and sense of shame experienced by Chinese MSM [5], [6]. The 5-to 6-fold increased risk of bipolar disorder found in the present study is consistent with findings in the Netherlands [8]; a congenital factor may contribute to such a high-level increase [36].

In contrast to what was noted when anxiety and mood disorders were studied, Chinese MSM had 12-month and lifetime prevalence rates of alcohol use disorders similar to those of their counterparts in the United States [9, -29-31, 33] and the Netherlands [8], but the prevalence rates in non-gay regional males were also lower than those of the general male populations of Western countries [8], [17]. The 3-fold elevation in the 12-month and lifetime prevalence rates of alcohol dependence was higher than that of MSM in the Netherlands (AOR  = 1.5 and 1.4, respectively) [8] and the United States (AOR  = 1.3 and 1.4, respectively) [9], [37], and was also higher than the 1.5-fold excess reported in an meta-analysis of homosexual men [10], and the finding of the California Quality of Life Survey (ARR  = 2.34, 95% CI  = 1.38–3.98) [30]. The higher risk for alcohol dependence in Chinese MSM is partly related to the relatively low prevalence of this condition in regional urban males, one possible explanation might be that Chinese MSM may be more likely to release or repress the stress or anxiety associated with their same-sex sexuality via alcohol abuse though we do not have empirical data to test this. Drinking is a very common mode of socialization in the gay community, especially when the so-called “alcohol culture” of China is considered [38].

As was seen when any disorder was studied, the 12-month and lifetime comorbidity rates for Chinese MSM (5.6% and 15.4%, respectively) and urban males (1.6% and 3.2%, respectively) were significantly lower than those of their counterparts in the Netherlands [8] and the United States [37]. Chinese MSM were at a 4-fold excess risk of 12-month (SPR  = 4.0, 95% CI  = 3.0–5.1) and lifetime (SPR = 4.2, 95% CI = 3.4–5.1) comorbidity, which is higher than the 2-fold elevation of comorbidity among MSM noted in the Netherlands [8], reflecting the larger disparity in comorbidity between Chinese MSM and urban males.

The present study had limitations. We used a relatively old version of CIDI1.0, which creates DSM-III-R diagnoses, rather than DSM-IV diagnoses. Differences in diagnostic criteria between DSM-III-R and DSM-IV may reduce the comparability of the data of the present study with those of some recent reports that employed CIDI3.0 to generate DSM-IV criteria. However, because the primary goal of the present work was to assess if MSM had an elevated prevalence of psychiatric disorders compared with regional urban males, and, if so, what such disorders were, it may have been appropriate to use CIDI1.0 because the present authors conducted a mental health survey in 2004 employing the same instrument [16], and several large-scale population-based studies have also employed CIDI1.0 [8], [9] to study MSM in Western countries. Thus, use of CIDI1.0 may enhance the comparability of both Chinese and international studies. We may have underestimated the prevalence of psychiatric disorders by combining mood, anxiety, and alcohol abuse disorders. Because the NCS criterion of “Any NCS disorder” and the NEMESIS criterion of “One or more DSM-III-R disorders” were used to compare MSM and control urban males in the present study, we suggest that our comparison was valid. Considering the low prevalence of other psychiatric disorders, we expect that the extent of underestimation of the prevalence of disorders will be small. In line with the NEMESIS criteria, we defined MSM as those who had had oral or anal sexual intercourse with another man in the past 12 months. Exclusion of gay subjects who were not sexually active tends to overestimate the prevalence of non-gay males; celibate gay subjects may have a higher prevalence of mental disorders than heterosexual males. Various studies have recorded discrepancies between homosexual behavior, homosexual orientation, and homosexual self-labeling. It would be valuable if future surveys incorporated both behavior- and identity-based definitions of sexual orientation. This would allow researchers to investigate the impact of discrepancies between these two definitions on the association between sexual orientation and mental health. We may have underestimated differences between MSM and heterosexual males because MSM were included in the general urban male population. However, if the 3.5% of MSM among the general male population are excluded, the SPR for the prevalence of any psychiatric disorder increases from 2.82 (95%CI: 2.47–3.21) to only 2.94 (95%CI: 2.57–3.34). Thus, the influence of the inclusion of MSM on SPR is negligible. Although RDS has been widely used to estimate the characteristics of hard-to-reach populations, such as MSM, the utility of RDS in real-life situations remains largely unknown; caution is required when interpreting RDS findings. The 807 MSM were selected from four cities of north-east China. It is not certain that our findings can be generalized to other regions of China. More studies are warranted.

According the knowledge of the author, the present study was the first to employ RDS to assess the prevalence of psychiatric disorders in MSM, using DSM criteria, which reduced the sampling bias evident in some surveys that employed conventional sampling methods, and the very high costs of population-based surveys. Our large sample size permitted analysis of both lifetime and 12-month prevalence of most disorders.
